# Health-Related Social Needs Discussions in Primary Care Encounters in Safety-Net Clinics: A Qualitative Analysis

**DOI:** 10.1001/jamanetworkopen.2025.1997

**Published:** 2025-03-26

**Authors:** Elaine De Leon, Sneha Panganamamula, Antoinette Schoenthaler

**Affiliations:** 1 Department of Medicine, NYU Langone Health, New York, New York; 2 Institute for Excellence in Health Equity, NYU Langone Health, New York, New York

## Abstract

**Importance:**

Health-related social needs (HRSN) influence health outcomes and health care utilization. Clinicians face challenges addressing HRSN due to limited skills, expertise, and time. Further insight is needed on how patients and clinicians navigate HRSN in clinical encounters.

**Objective:**

This study examines outpatient primary care encounters predating widespread HRSN screening to identify how discussions on HRSN are initiated and addressed.

**Design, Setting, and Participants:**

This qualitative analysis was conducted on transcripts of 97 audiotaped English-speaking patient encounters from 3 clinics in New York City within a municipal health care system from January 2011 through April 2015. Patients were eligible if they were older than 18 years, self-identified as Black or White, had a diagnosis of hypertension, and had at least one prior encounter with the participating clinician. Codes were developed from social needs domains addressed by the Accountable Health Communities HRSN Screening Tool. Codes were added for further social needs identified, whether a patient or clinician initiated the HRSN discussion, and how a social need was addressed, if at all. Encounters were analyzed between June 2023 and February 2024.

**Main Outcomes and Measures:**

Characterization of the content and nature of HRSN discussions during clinical encounters within safety-net clinics.

**Results:**

A total of 97 patients (55 [56.7%] women, 58 [59.8%] Black, mean [SD] age, 59.7 [10.6] years) had audiotaped encounters with 27 clinicians (18 [66.7%] women, 15 [55.6%] White, mean [SD] age, 36 [5.8] years). Physical activity (36% of encounters), financial strain (35%), mental health (34%), and substance use (28%) were the most discussed HRSN domains across the 97 encounters. Patients introduced financial strain most often (70% of the time), while clinicians led substance use (75%), physical activity (51%) and mental health (51%) discussions. Patients initiated conversations on employment (77%), food insecurity (62%), and housing instability (52%). Interventions included prescriptions, forms, counseling, and referrals. Domains frequently intervened on included health care navigation needs (85% of discussions), substance use (33%), and mental health (27%).

**Conclusions and Relevance:**

In this qualitative study of HRSN discussions in primary care encounters, clinicians were more likely to initiate discussions on substance use, physical activity, and mental health, behaviors routinely assessed in primary care, but different from topics introduced by patients. Findings underscore the need for standardized screening to improve identification of domains less frequently addressed by clinicians. Additional interventions are also needed, including clinician training for how to address HRSN in resource-constrained settings and integration of other health care team members, to enhance HRSN identification and intervention.

## Key Points

**Question:**

In the absence of standardized screening for health-related social needs (HRSN), how are HRSN discussed and addressed during primary care encounters in safety-net clinics?

**Findings:**

A qualitative analysis of 97 audiotaped patient encounters found that the HRSN domains of physical activity, financial strain, mental health, and substance use were commonly discussed. Patients and clinicians differed with regard to which domains they introduced during encounters.

**Meaning:**

Findings highlight the utility of standardized screening and need for targeted interventions, such as training and integration of other health care team members, to improve comprehensive identification of social needs in resource-constrained settings.

## Teaser

This qualitative analysis characterizes the types of screening and referral discussions around health-related social needs that occur within outpatient primary care visits conducted in urban safety-net clinics.

## Introduction

The unequal distribution of the social determinants of health (SDOH) leads to health-related social needs (HRSN) that impact health outcomes^1,2,3,4,5,6^ and health care utilization.^1,2,7,8^ Identifying and addressing HRSN is integral to reducing health disparities.^9,10,11^ Various federal initiatives are being implemented to promote and standardize HRSN collection.^12,13,14,15,16^ These efforts will strengthen our understanding of how SDOH impact clinical outcomes. There is currently no standard method for HRSN screening, although many tools have been adopted by health systems,^17^ including Centers for Medicare and Medicaid Services’ Accountable Health Communities (AHC) HRSN Screening Tool.^18^

Identifying and addressing patient HRSN creates greater alignment between patient and clinician priorities,^19,20^ preventing delays in care.^21,22^ Despite strides to integrate social care into standard practice across health care settings,^23,24,25,26^ both patient and clinician-level challenges hinder HRSN screening and referral. Patient-level barriers include confusion around the connection between HRSN and health and concerns for stigma and data privacy.^24,27,28,29,30^ However, literature suggests most patients support HRSN screening in health care.^29,31,32,33^ Within primary care, competing demands on time, differing levels of training, growing levels of burnout, lack of referral resources, and uncertainty around whose role it is to screen and refer are all factors contributing to clinician perceptions in screening acceptability and sense of comfort and competence.^29,34,35,36,37,38,39,40^ Conversely, facilitators to screening include having dedicated staff for screening^31,41^ and integration into the electronic health record.^42,43^

Beyond broad understanding of what influences screening and referral practices in health care settings, there is limited literature examining which HRSN domains are commonly addressed, who initiates the conversation, and how such matters are addressed. Through content analysis of outpatient primary care visits conducted in urban safety-net clinics, we aim to provide novel insights on discussions around HRSN to characterize the types of screening and referral discussions that occur within resource-limited health care settings.

## Methods

This study utilized a qualitative content analysis approach on a collection of audiotaped clinical interactions between primary care clinicians and their patients. Transcripts were sourced from a prior study investigating patient-clinician communication and medication adherence among Black and White individuals with hypertension.^44^ This study adheres to the Standards for Reporting Qualitative Research (SRQR) reporting guideline.

### Study Design and Participants

Patients and clinicians were recruited between January 2011 and April 2015 from 3 primary care clinics within one large municipal health system in New York City that serves as a major source of primary and hospital-based care for a diverse range of patients regardless of ability to pay. Patients were eligible if they were over 18 years old, had a hypertension diagnosis, and had at least one prior visit with a participating clinician. Because transcripts were drawn from a study examining differences between Black and White individuals in patient-clinician communication, patients from other racial or ethnic groups were not represented, although clinicians separately identified as Asian, Black, Hispanic, or White. Patients were recruited and consented to the study in clinic prior to visits while clinicians were recruited and consented during a staff meeting. Written informed consent was obtained from all participants allowing use of deidentified audiotapes for any research and training purposes, including publication of quotations, with no time limit on their use. Identity characteristics of study participants are reported by race and ethnicity (Asian, Black, Hispanic, and White), sex (female or male), and participant role (patient or clinician). This study was approved by NYU Langone’s Institutional Review Board. Encounters were analyzed between June 2023 and February 2024.

### Data Collection

Encounters were audiotaped via a recorder placed in the examination room by a research assistant. Participants were instructed that they could turn off the recorder at any time. Recordings were deidentified and professionally transcribed. Prior to each visit, a survey was used to capture clinician and patient age, sex, ethnicity (Hispanic/Latino vs Not Hispanic/Latino), and self-reported race among many other characteristics. Demographic data and transcripts were uploaded to a mixed methods research software (Dedoose; SocioCultural Research Consultants, LLC; version 9.0.17) for analysis. No formal social needs screening was conducted during the data collection period; therefore HRSN discussions were unprompted.

### Data Analysis

A codebook was created based on the 5 primary social needs (ie, housing instability, food insecurity, transportation, utility help needs, and interpersonal safety) and 8 secondary social needs (ie, financial strain, employment, family/community support, physical activity, substance use, mental health, disability, and education) outlined in the AHC HRSN Screening Tool. For the physical activity domain, coders excluded conversations related to impairment due to chief concern, physical therapy, or activity necessitated by a test (eg, stress test).

The transcripts were initially read and deductively coded based on the codebook of HRSN domains. Inductive codes were added for needs identified not explicitly delineated in the screening tool (eg, health care navigation, difficulty paying for health care), whether a clinician or patient initiated the HRSN discussion, and how a clinician intervened on a social need, if at all (eg, direct assistance or providing a referral). These codes were applied on a second reading of the transcripts. Transcripts were independently read and coded by two analysts (E.D. and S.P.) trained in qualitative methods who are primary care clinicians. ED identifies as a Black Hispanic woman and SP as a South Asian woman. Consensus on the codes applied was reached through shared review of all excerpts to ensure agreement with categorization before identifying themes by HRSN domain.

## Results

A total of 97 patients (55 [56.7%] women, 58 [59.8%] Black, mean [SD] age, 59.7 [10.6] years) had audiotaped encounters with 27 clinicians (18 [66.7%] women, 15 [55.6%] White, mean [SD] age, 36 [5.8] years) (Table 1 and Table 2). Our findings correspond to the primary and supplemental domains of the AHC HRSN Screening tool followed by inductive codes. Educational level was not addressed in the encounters. Table 3 provides a full list of domains with corresponding representative quotations and intervention examples. We then explored patient initiation compared with clinician initiation of HRSN discussions.

**Table 1.** Patient Characteristics

Characteristic	Total sample, No. (%) (N = 97)
Age, mean (SD)	59.7 (10.6)
Gender	
Male	42 (43.3)
Female	55 (56.7)
Race^a^	
Black	58 (59.8)
White	39 (40.2)
Marital status	
Single	40 (41.2)
Married	17 (17.6)
Divorced/separated	27 (27.8)
Widowed	13 (13.4)
Educational level	
<High school	11 (11.3)
>High school	86 (88.7)
Employment status	
Employed	32 (33)
Unemployed	43 (44.3)
Retired	22 (22.7)
Annual income	
<$10 000	23 (23.7)
$10 000-40 000	41 (42.3)
$40 000-100 000	10 (10.3)
>$100 000	3 (3.1)
Did not disclose/other	20 (20.6)
Insurance status	
Medicaid	36 (37.1)
Medicare	25 (25.8)
Medicaid and Medicare	4 (4.1)
None	18 (18.6)
Private	14 (14.4)
Years with primary care professional	
<1 y	34 (35.1)
1-5 y	34 (35.1)
>5 y	29 (29.8)

^a^ Because transcripts were drawn from a study examining differences between Black and White individuals in patient-clinician communication, individuals from other racial or ethnic groups were not represented.

**Table 2.** Clinician Characteristics

Characteristic	Total sample, No. (%) (N = 27)
Age, mean (SD)	36 (5.8)
Years in practice, mean (SD)	5.8 (4.6)
Gender	
Male	9 (33.3)
Female	18 (66.7)
Race	
Asian, not Hispanic/Latino	5 (18.5)
Black, not Hispanic/Latino	5 (18.5)
Non-White, Hispanic/Latino	2 (7.4)
White, Hispanic/Latino	1 (3.7)
White, not Hispanic/Latino	14 (51.9)
Title	
MD	26 (96.3)
NP	1 (3.7)

Abbreviations: MD, doctor of medicine; NP, nurse practitioner.

**Table 3.** Representative Quotations and Clinician Intervention Examples by Domain

Social need domain (No. of encounters)	Representative quotation (participant)^a^	Intervention example (participant)
Housing instability (10)	“This experience of being homeless has taken a toll on me... so I’ve unraveled.” (Black male patient)	Clinician: I can definitely fill out this form. Patient: Basically, let them know that I need to go some place where senior people live…I really appreciate any help you can give me, giving any medical reasons. (Direct assistance; Black male patient, Asian female clinician)
	“So where are you living? Back with your niece in New Jersey? Are you back here?” (Hispanic female clinician)	
Food insecurity (11)	“I try to eat healthy, but I work on the street. There’s nothing. It’s garbage.... It’s expensive to eat healthy. And things that are not good for you are like $2.” (Black male patient)	NA
	“[Have you lost weight] on purpose, or is that because you’re not getting enough food?” (White female clinician)	
Transportation problems (4)	“I walk basically most places I have to go…I’m not [taking] too much buses. I mean, I can’t afford all that transportation.” (Black female patient)	Patient: I don’t know if you do this or not…. they took away my Access-A-Ride and I’m fighting it.... Clinician: So what I can do is I can write a letter for you to take. (Direct assistance; Black female patient, Black female clinician)
Utility help needs (4)	“I should give you another number because ... my subsidized phone is tied to the, uh, my Medicaid coverage …” (White female patient)	NA
Interpersonal safety (2)	“I’m wondering what’s gonna happen if we have a physical confrontation? Last time that happened, I wind up doing the 18 years.” (Black male patient)	NA
	“Is he ever mean to you or get aggressive or violent or anything?” (White female clinician)	
Financial strain (34)	“It was 150 just for an MRI and a colonoscopy. So how much would it be for surgery?... I can already tell you it’s going to be beyond what I can afford on unemployment.” (Black female patient)	Patient: I still have a couple packets left. I need to use that up before I get more. Clinician: That’s what you think or that’s what your pharmacy told you? Patient: No, that’s what my pocketbook told me. Clinician: Okay. I understand. It’s a very expensive medication...Well, you know what the other thing you could do? Right now, you do one puff in the morning and one puff at night, right? … You can do two in the morning and two [in the evening]. And that will be the same. (Counseling; Black female patient, Hispanic female clinician)
	“I do have a lot of patients that can’t afford [medications] so they take them five days a week instead of seven days a week…that’s not an issue for you?” (White female clinician)	“I’ll get you to talk with a social worker. So that they’ll help you deal with this.” (Referral; Black male clinician)
Employment (12)	“I tried to get on welfare and they’re giving me a hard time. They want me to go back to work … I said, ‘There’s no way I can go back to work. I got too many health problems’.” (White male patient)	Patient: I need a letter saying I was seen in the clinic today. Clinician: Yeah, no problem. We’ll give you a letter. For what? Patient: I’m in [name of program]. (Direct assistance; White male patient, Black female clinician)
	“Are you working still?.... Do you have a day off during the week?... Monday is your day off. If the CAT scan was scheduled for a Monday, would that work for you?” (White female clinician)	
Family and community support (17)	“That extended family and you will just call and you would talk that, oh, your diabetes, blah, blah, blah… And then you will in conversations say, ‘How is it going?’ But here in the United States, the extended family is not that great. Especially in the big cities... you’re mainly in an apartment… there’s no aunts and uncles that you can see.” (Black male patient)	“It may not be for many years that you need the help, but just check into it.... There’s people that come and help with, like, bathing and that kind of stuff. And there’s also care where people can come and give you a little break so you can go out.” (Counseling, White female clinician)
	“You got somebody who you talk to when you get stressed?” (White female clinician)	
	“My granddaughter was just [saying], ‘Grandpa, you’re going down to do a cigarette?’ … ‘Stop doing the [cigarettes].’” (White male patient)	
Education (0)	NA	NA
Physical activity (35)	“You know, other than that…and yoga. I found two classes I can take. So one of the perks of having breast cancer is you can access some things so…there’s one for five dollars…one for free.” (White female patient)	“Your everyday activity is not enough to burn enough calories for you to lose weight, unless you happen to be a construction worker or, you know, a dock worker.... So we recommend, let’s say four times a week, about 40 minutes, moderate physical activity. Now for you, a guy who doesn’t run or doesn’t do anything else, that in the beginning will simply be brisk walk. So I’m not even asking you to run... just walk.” (Counseling, White male clinician)
	“The last time I saw you we set a goal of taking a 10 or 15 minute walk most days.” (White female clinician)	
Substance use (27)	“I grew up knowing about smoking before I started. I don’t have the ignorance excuse. I smoked because I was really angry. I mean, when I say really angry I mean think bonfire.... I felt like it didn’t matter.” (White male patient)	“If you decide to go back on the patch if things are getting tricky and you’re smoking more, consider not using the full 21. Maybe try the 7. It roughly correlates with how many cigarettes you’re having… If you’re having half a pack a day, then the 14. If you’re having a full pack, it’s the 21.” (Counseling, Hispanic female clinician)
	“What am I gonna do about having you quit smoking, now? Do you want me to give you a pill, a patch, a gum?” (Black female clinician)	
Mental health (33)	Patient: I was depressed that’s why I wasn’t taking my high blood pressure medications properly. Then when you spoke with me and then you helped me decide that we’ll just try the depression medication. Then once I took the depression medication, I realized it was just being depressed that wasn’t allowing me to take my medicines every day, but now I do.... I’m just happy you were able to talk with me about that. Clinician: Yeah. I know losing your son was very difficult. (Black female patient, White male clinician)	“It seems like you’re coping well, but if ever you’re not coping well, please come to the emergency room.... There’s always a psychiatrist there just to help you with, like, managing and coping, ’cause it sounds like you’re under a lot of stress.” (Counseling, Asian female clinician)
	“It’s okay to be difficult, but it’s also okay to have help with your feelings as well, and I’m glad to see that we’re finding a way to help you with your feelings.... Great, so some questions about your feelings. In the last two weeks, how often have you felt little interest or pleasure in doing things that you normally like to do?” (White male clinician)	“Let me give you a medication for depression, even though you don’t really fit the classic description.” (Direct assistance; White female clinician)
Disabilities (12)	“It never was this hard to really ambulate myself out of bed. And that’s worrying me ’cause it’s affecting my everyday movement. It’s also affecting my exercising … And that frustrates me ’cause I want to be active and keep on the road to my goal of losing weight and healthier lifestyle.” (Black female patient)	Patient: Now what I do need is to see the social worker. Because I need help. I need a home attendant. And she told me that you have to fill out a 10 something. Clinician: M11Q. She fills it out. I sign it…That’s a great idea. I’ll set that up. (Direct assistance; Black female patient, White male clinician)
	“Number two is just keep track of if you start falling and stuff, we’re gonna have to even work on something better than even this walker.... Then the third thing is the driving. You sound like you’re doing great with the driving.... If you notice that you’re getting into bad situations, you gotta say something.” (White male clinician)	
Healthcare navigation (15)	“Hi... I’m calling from the Medicine Clinic. I wanted to see if I could facilitate one of my patients getting an outpatient CT…They were closing up downstairs, so I wanted to call for her.” (White female clinician)	“Let me see if they actually made the appointment… If they didn’t make the appointment yet, I can have my assistant, when you check out now, I can have them make it for you.” (Direct assistance; Hispanic female clinician)

^a^ Representative quotations are presented verbatim but have been lightly edited to remove filler words (eg, um, you know) for clarity and readability without altering meaning.

### Primary Domains

#### Housing Instability

Discussions around housing instability detailed patient experiences with the shelter system, harmful exposures within housing environments (eg, fumes from building renovations), and limited access to appliances. Two patients reported experiencing homelessness, one reporting dissatisfaction with their shelter, “I left the shelter system and lived in the street for two weeks. I got mad with them.... The only reason I came back is ’cause my leg started swelling up” (Black male patient). Across encounters, counseling or referrals for housing assistance were not offered, although a patient received housing application paperwork following their request.

#### Food Insecurity

Patients did not report difficulty obtaining food but faced significant challenges in reliably accessing healthy food due to cost concerns, geographic availability (eg, while at work), or dislike for what they were served. Clinicians did not counsel on less expensive healthy options, nor did they refer patients to social services.

#### Transportation Problems

Transportation discussions focused on difficulties with paratransit services for those with handicaps that prevent use of public transportation. This service was implicated in delays in getting to medical appointments. Despite the service’s lack of reliability, patients depended on it and requested clinician assistance with letters to maintain it.

#### Utility Help Needs

Utilities discussions were limited to challenges with phone access and a water bill. Regarding phone access, patients brought up concerns when clinicians would ask for contact information. In one encounter, the patient reported their phone minutes were tied to their health insurance.

#### Interpersonal Safety

Discussions of interpersonal safety largely pertained to a patient who had frequent interactions with police and the criminal justice system. The clinician displayed empathy for the patient, although resources to legal recourse were not provided. In another encounter, a patient who was caregiver to an older adult with dementia was screened for interpersonal violence.

### Supplemental Domains

#### Financial Strain

Financial strain was frequently discussed, typically among patients having trouble affording medical care due to underinsurance: “Patient: I’m scared I’m gonna run out of my medicines and can’t even afford to buy them.... I need my atenolol and I don’t know how I got so many pills that I have to take and I gotta get renewed.... I just have to go and see which ones are the ones that I need the most” (Black female patient).

In response, clinicians modified treatment regimens to more cost-friendly options, recommended delay of care during hardship, completed public assistance paperwork, or wrote letters of medical necessity. Patients also, although less frequently, expressed the inability to afford healthy food, transportation, and utilities. Only one referral to social work was made in the context of a patient’s excess medical bills.

#### Employment

Employment was inextricably linked to health and health care across the encounters where the domain was discussed. Poor health status restricted patients’ ability to seek and maintain stable or higher-earning employment. Unfortunately, patients’ dependence on employer-sponsored insurance impeded access to the care necessary to improve their health. For example, one patient awaiting hip surgery to reenter the workforce also unable to afford the procedure described this no-win situation: “I have no insurance, so [hip surgery’s] out until I can find a job with insurance … I don’t qualify for [Medicaid] … I made too much on unemployment” (Black female patient).

#### Family and Community Support

Family and community support was often addressed in the context of rapport building but also to identify individuals that patients felt able to rely on or assist with medical care and decision-making. Family and other support members were trusted to provide collateral information to clinicians. The presence of family and/or community support was expressed as a motivator, and the absence of family and/or community support was expressed as a barrier, of completing health-related tasks including the development of healthy behaviors and making appointments.

#### Physical Activity

Discussions of physical activity often co-occurred with discussions of diet, demonstrating that patients and clinicians linked the two activities in efforts to address chronic conditions. Clinicians usually provided counseling regarding physical activity, although this intervention was often limited to recommendations on duration. When addressing physical activity, there was little discussion on where patients were able to exercise or whether they felt safe exercising with only one encounter highlighting the built environment: “Bloomberg fixed the water in Manhattan where you could walk... along the FDR Drive.... I walk from 145th all the way to 131st Street in laps” (Black female patient).

#### Substance Use

Most discussions on substance use involved clinicians screening for use disorder, most frequently regarding tobacco or alcohol. Questions were typically weaved into conversations: “How [are] things going with the alcohol?” (Black female clinician). There were also instances of clinicians inquiring about substance use to inform management decisions (eg, clinician who did not prescribe an opiate to a patient with a history of a use disorder).

Substance use discussions most frequently resulted in patients denying history of use. In cases of continued tobacco or alcohol use, clinicians intervened with medications, motivational interviewing and/or counseling: “How about a medication that decreases the cravings?” (White female clinician). No encounters featured counseling or referrals to opioid treatment and there were limited inquiries or counseling regarding prescription misuse.

#### Mental Health

Mental health discussions were common although varied in focus depending on whether the conversation revolved around stress and life events vs the medical management of mental health conditions. Stress management was recommended by clinicians to improve both mental and physical health: “I think that one way to help yourself feel better is just to … try to figure out a way to manage that stress” (Asian female clinician). Discussions around mental health conditions focused on medical management, with clinicians emphasizing medication effectiveness for improved mood:

Clinician: Last time we talked about the fact that maybe it might be good for you to get started on a medicine to help you with this problem of being depressed.... Does that sound okay with you?

Patient: Sounds fine.

Clinician: Alright, so, we’ll take care of that problem with a medication” (White male patient; Hispanic male clinician).

In a few cases, clinicians suggested speaking with psychologists or counselors to address patients’ mental health concerns and provide an outlet to “just kind of get things off [their] chest” (Hispanic female clinician).

#### Disabilities

Disabilities were typically brought up in the context of a chief concern that was causing temporary disability, reliance on assistive devices (eg, canes), or employment challenges due to chronic disability. Conversations did not usually address the impact that disabilities may have on health-related quality of life. Patients expressed frustrations about disabilities hindering their ability to secure employment due to discrimination or limitations imposed by their condition: “It’s like they don’t have to discriminate against me and say outward, no, we won’t hire you ’cause... you have a disability, but there’s a way getting around it” (Black female patient). Patients with these challenges asked clinicians for assistance in applying for disability benefits.

#### Health Care Navigation

**Figure 1.**
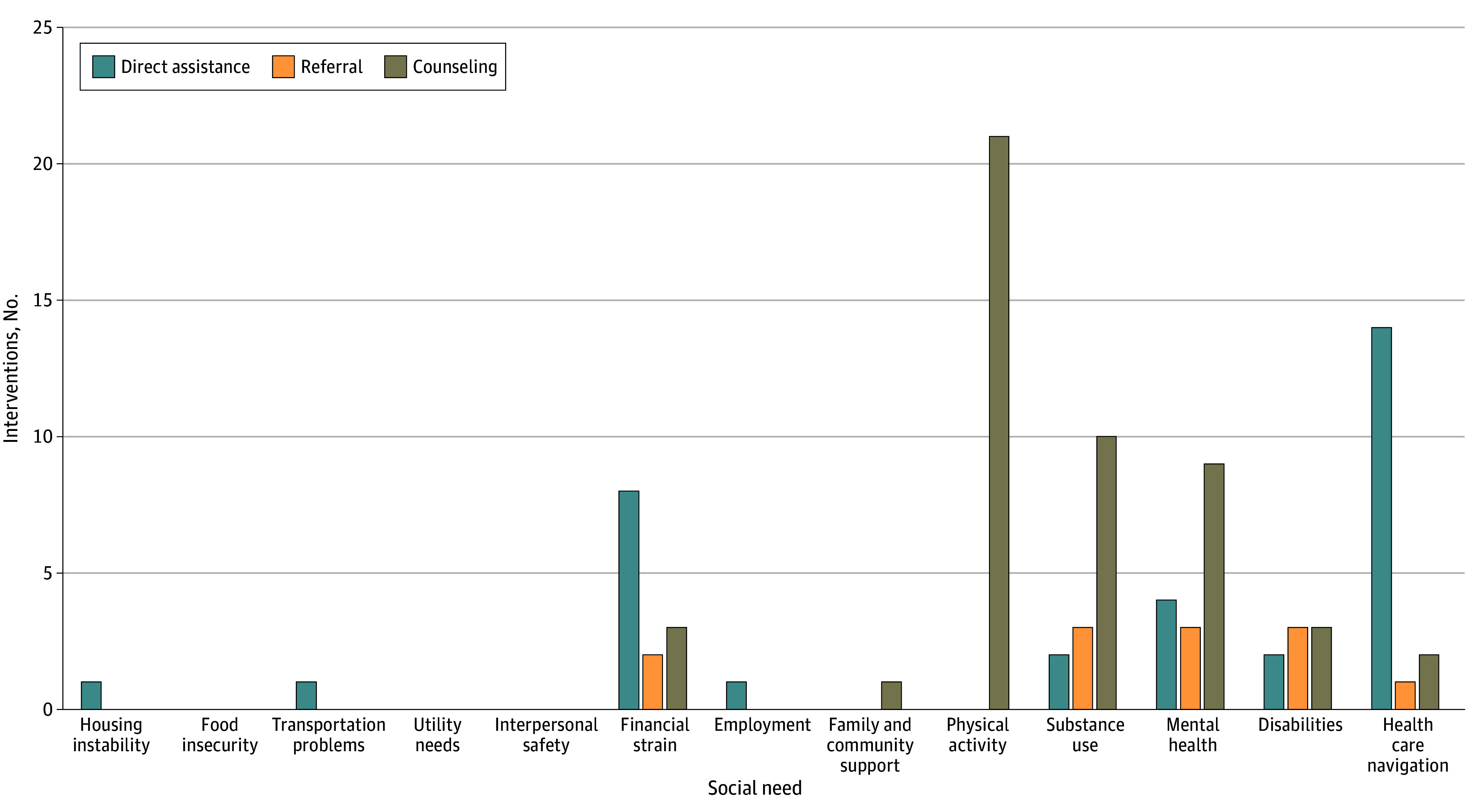
Social Need by Number and Type of Clinician Intervention

Health care navigation, although not an HRSN, represented an inductive code reflecting challenges patients faced in navigating the health care system. Patients requested or were helped in scheduling appointments, printing future appointment and medication lists, and addressing challenges in filling prescriptions. Assistance with health care navigation was the most frequent need that was intervened on during patient encounters (85%), followed by the domains of substance use (33%) and mental health (29%) (Figure 1).

### Patient and Clinician Initiation of HRSN Discussions

**Figure 2.**
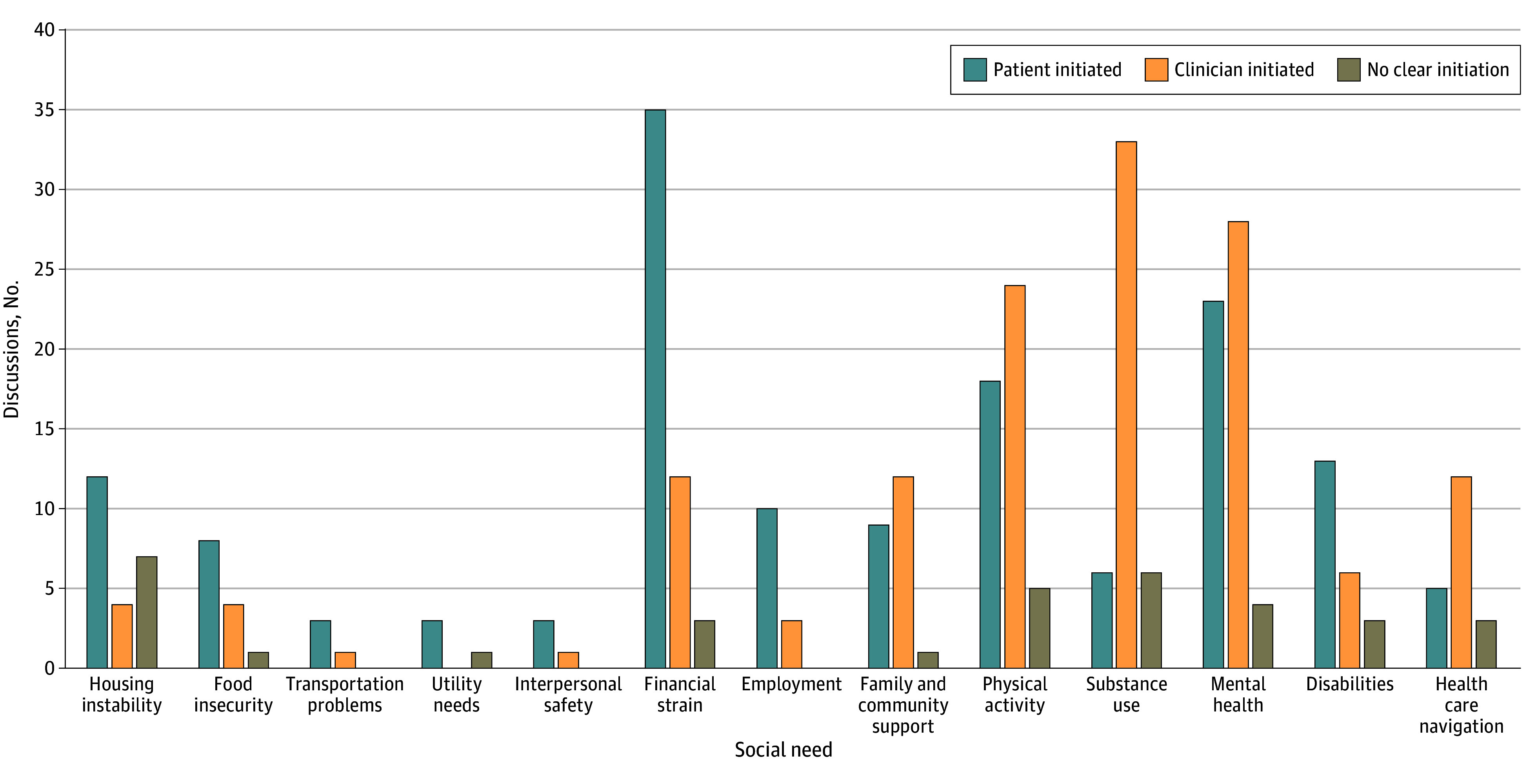
Social Need Discussed by Patient or Clinician Initiation

Domains differed by whether patients or clinicians initiated discussion around HRSN topics (Figure 2). Conversations around financial strain were initiated by patients 70% of the time, whereas conversations regarding substance use were initiated by clinicians 73% of the time. Patients more frequently initiated discussions on employment (77%), food insecurity (62%), and housing instability (52%). Meanwhile, physical activity was initiated more often by clinicians than patients (51%vs 38%), and mental health discussion were also initiated more often by clinicians than patients (52% vs 42%). HRSN domains that were less likely to be discussed by either patients or clinicians included education (0% of encounters), interpersonal safety (2%), utility needs (4%), and transportation (4%).

Black patients introduced HRSN more commonly than White patients (59% vs 41%) and male patients were slightly more likely to bring up these needs compared with female patients (52% vs 48%). Black patients were equally as likely to initiate HRSN discussions as their clinicians, but clinicians were more likely to initiate HRSN discussions with White patients (56% vs 44%).

## Discussion

Our investigation explored discussions on HRSN in 97 audiotaped outpatient primary care encounters for patients with hypertension. These conversations predated the widespread recognition of and federal mandates for HRSN screening.^45^ Physical activity, financial strain, mental health, and substance use emerged as the most frequently discussed domains, each addressed in approximately one-third of encounters. All social needs discussed had important linkages to patients’ health and well-being as well as ability to engage in the prevention and management of medical conditions.

Compared with patients, clinicians more frequently initiated discussions on substance use, physical activity, and mental health, offering interventions more often for these domains. These domains may potentially be perceived as more easily addressed by clinicians within resource-strained settings and within the scope of their professional training. Although prior survey data from the Physicians Foundation identified that clinicians believe that financial instability and transportation concerns are the top two social needs that patients experience,^46^ across all encounters, conversations on financial strain and transportation needs were typically patient-initiated. Patients were also more likely to bring up housing instability, food insecurity, employment, and important challenges in their ability to afford medications. Clinicians were about half as likely to ask about medication affordability despite the tacit expectation that patients should adhere to medication regimens.

Our findings also identified that Black patients in the sample were more likely to initiate HRSN discussions compared with White patients. Other studies have identified additional patient-level factors that increase integration of HRSN into routine care, with male gender and non-English language increasing the likelihood that SDOH influence care.^42^ Patients with frequent clinic visits or who have multiple comorbidities also tend to be screened for HRSN more frequently.^47^

The discrepancies between clinician-addressed and patient-addressed domains and racial differences in initiating HRSN discussions identified in this analysis underscore the importance of implementing standardized HRSN screening across health systems. However, these efforts must incorporate strategies for how patient perspectives can play more central roles in shaping encounters. HRSN data provided through formal screening tools should be complementary and not a replacement for discussions between patients and clinicians.^42^ To align patient and clinician agendas, previous work recommends health care teams use a relationship-centered framework to address SDOH.^19^ Within this framework, attention is paid to interpersonal communication among care team members and the respectful framing and partnership conveyed to patients during the screening and referral process.^19^

Alignment at the patient and care team levels will not happen organically,^48^ even with federal mandates for HRSN screening. Although clinicians feel a sense of responsibility to incorporate social care into clinical practice, feasibility remains a major concern. Clinicians may not feel empowered to meaningfully assist patients with HRSN due to time constraints or lack of support, particularly in resource-strained settings.^49^ Strategies to improve screening could include comprehensive clinician training,^26,37,49^ Electronic health record–integrated HRSN screening and referral platforms,^50,51,52^ and leveraging team members such as community health workers to facilitate linkages to community-based services.^53^

### Limitations

Our findings must be considered in the context of our study’s limitations. Encounters focused on English-speaking patients of Black or White race in 3 municipal clinics in New York City, which may limit generalizability. The exclusion of non-English speakers introduces potential language biases, highlighting the need to explore how HRSN are discussed when language barriers are present. The safety-net clinic clinicians are likely more accustomed to addressing social needs, and the urban environment may not reflect the experiences of patients in more resourced health care settings and/or in rural areas. We also did not include perspectives from clinicians, patients, or other team members, suggesting a need for future qualitative studies. Additionally, relying on audiotaped encounters limited our observation of nonverbal behaviors. Despite these limitations, our study offers valuable insights on the nature of and intervention on HRSN in outpatient primary care.

## Conclusions

In the absence of mandated social needs screening, our qualitative analysis found that patients and clinicians in safety-net clinics were able to discuss and address HRSN. However, there were differences in the domains introduced by patients vs clinicians, reinforcing the need for standardized screening and targeted interventions to facilitate these discussions. Overall, we need a nuanced approach to HRSN screening that will account for clinician biases and patient priorities in diverse health care settings. Moreover, our approach must support both patients and clinicians as they navigate through these essential yet challenging conversations.

## Notes

Supplement.
Data Sharing Statement
