# Supplementary material for: Health-Related Social Needs Discussions in Primary Care Encounters in Safety-Net Clinics: A Qualitative Analysis
Source: JAMA Netw Open. 2025 Mar 26;8(3):e251997. doi: 10.1001/jamanetworkopen.2025.1997 (PMC11947842; doi:10.1001/jamanetworkopen.2025.1997)
Supplement: Supplement. — Data Sharing Statement [file jamanetwopen-e251997-s001.pdf]

Data Sharing Statement

De Leon. Health-Related Social Needs Discussions in Primary Care Encounters In Safety-Net Clinics. *JAMA Netw Open*. Published March 26, 2025.
doi:10.1001/jamanetworkopen.2025.1997

Data
Data available: No
